# Highly efficient eco-friendly sodium titanate sorbents of Cs(i), Sr(ii), Co(ii) and Eu(iii): synthesis, characterization and detailed adsorption study†

**DOI:** 10.1039/d3ra05663e

**Published:** 2024-01-05

**Authors:** Monika Motlochová, Lórant Szatmáry, Eva Pližingrová, Petra Salačová, Radek Fajgar, Sven Lidin, Jan Šubrt

**Affiliations:** a Institute of Inorganic Chemistry of the Czech Academy of Sciences 250 68 Řež Czech Republic motlochova@iic.cas.cz; b Centre for Analysis and Synthesis, Lunds Universitet Naturvetarvägen 14 Lund 222-61 Sweden; c Fuel Cycle Chemistry Department, ÚJV Řež a.s. 250 68 Řež Czech Republic lorant.szatmary@ujv.cz; d Institute of Chemical Process Fundamentals of the Czech Academy of Sciences Rozvojova 135 Prague 160 00 Czech Republic

## Abstract

Development of useful all-around materials which can quickly and efficiently adsorb radionuclides in response to environmental radioactive contamination is an urgent research objective. In response to this need, our team developed a simple preparation method for stable sodium titanates which can serve as efficient agents for removal of radionuclides from water. With an emphasis on an environmentally friendly synthesis, the resulting materials were defined by a range of means and methods measuring *e.g.* pH, ionic strength, contact time or metal ion concentration in order to assess their potential for use and applications as sorbents. The data obtained from measurements revealed rapid removal kinetics (up to 10 minutes), wide range of pH use and high equilibrium capacity. The maximum amount of adsorbed ions as calculated from the Langmuir isotherm was equal to 206.3 mg g^−1^ for Cs(i), 60.0 mg g^−1^ for Sr(ii), 50.2 mg g^−1^ for Co(ii) and 103.4 mg g^−1^ for Eu(iii), significantly exceeding published data obtained with related materials. The removal mechanism is most likely ion exchange followed by complexation reactions, as indicated by TEM/EDS analyses. Given their extraordinary sorption capacity and facile synthesis under mild conditions, these materials are promising candidates for the efficient removal of radionuclides from aqueous solutions during the clean-up of radioactive pollution in the environment.

## Abstract 2

A novel way of simple preparation of sodium titanates was described and their ion-exchange mechanism was studied for the removal of Cs(i), Sr(ii), Co(ii) and Eu(iii) from aqueous environments.

## Graphical abstract

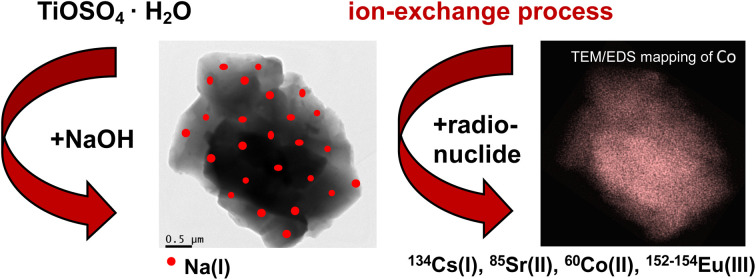

## 1. Introduction

Liquid wastes containing non-ferrous heavy metal ions and some radionuclides (^137^Cs and ^90^Sr in particular) represent potentially one of the most dangerous sources of environmental contamination. The remediation of wastewater containing such pollutants continues to be amongst the biggest challenges of sustainable development and environmental safety. Sorption-based technologies have proved their efficiency in reducing the radionuclide content in aqueous streams to low-level residual activity, with the concomitant decrease in the generated amount of ultimate solid waste.^1,2^

Titanate based substances are known to be stable with respect to radiation, as well as chemical, thermal, and mechanical changes.^3,4^ Many solid nanostructured titanium oxide based materials showed excellent ability to absorb various radionuclides.^1^ Moreover, these compounds are characterized by their excellent ion-exchange capacity and fast sorption kinetics. Recent studies have also been focused on layered materials, *i.e.* layered titanate structures in different morphologies: nanotubes, nanowires, nanofibers or even nanoribbons.^5–10^ Effective radionuclide sorption was also observed with silica-titanates.^11^

The presence of different radioactive elements in water, *e.g.* Cs^+^, Sr^2+^, Eu^3+^ and Co^2+^ is of major concern because of their high toxicities and carcinogenic effects on human.^12^ Therefore, the accidental release of large amounts of radioactive elements into the aquatic environment has become a direct threat for human health.^12,13^ All these radionuclides are very hazardous because of their high solubility, high transferability and easy assimilation into living organisms.

Many physical and chemical methods have been proposed for the removal of Cs radionuclides from contaminated wastewater, such as solvent extraction, chemical precipitation, membrane processes, coagulation, electrochemical and ion exchange.^14,15^ Methods, such as solvent extraction, ion exchange and adsorption, could be widely used due to their ionic selectivity, and solvent extraction using the macrocyclic ligands such as crown ethers, calix-crowns and chlorinated cobalt dicarbollide can be initiated to remove these radionuclides.^16–18^ Sorption at the solid–liquid interface has attracted much scientific and technical interest mainly because of its high degree of control, flexibility, and ease of use together with either the reduction in the volume of solid waste to be stored in landfills or the reversibility of the sorption phenomenon allowing preservation of raw materials.

Titanate nanostructures are usually synthesized *via* a hydrothermal process. For this purpose, a precursor of titanium dioxide is mixed with a solution of NaOH or KOH to obtain a suspension, which is subsequently placed in a polytetrafluoroethylene autoclave under constant stirring at 200 °C for 3 days.

We have recently developed a new preparation method for the synthesis of hydrated titanate microrods in aqueous media starting with solid hydrated titanyl sulphate crystals with defined morphology. The particle size and morphology of the starting hydrated titanyl sulphate is closely preserved in the pseudomorphs of amorphous metatitanic acid or alkali obtained metatitanates including such details like the layered structure of the original hydrated titanyl sulphate crystals.^19,20^ The amorphous character of the material was documented by the electron diffraction pattern with only very broad signs of diffraction rings and by high-magnification image showing a porous character of the product.^19^ The rod-like particles of metatitanic acid possess excellent sorption properties toward heavy metals (*e.g.* Pb^2+^).^21,22^

Following the general demand for preparation of suitable adsorbents for radionuclides, the group efforts focused on suitable modifications of this material. In the present work, a sodium titanium oxide materials were synthesized in a fast and cost-effective way, the products were characterized by methods of electron microscopy, thermal analysis and surface area determination and then on the most suitable material, the effects of parameters such as contact time, pH, ionic strength and metal ion concentration were also investigated to assess the potential application of prepared titanium based sorbent in real applications.

## 2. Materials and methods

### 2.1. Synthesis

Based on alkaline controlled hydrolysis, a sodium titanium oxide was prepared according to the following procedure:^27,28^ a 100 ml of cooled distilled water was mixed with 50 g of ice and 0.07, 0.14 and 0.21 mol NaOH in form of a solution (50 g into 100 ml, Penta, Czech Republic), then 4.80 g of titanyl sulphate (monohydrate, provided by local supplier Precheza a.s.) was added. While the mixture was magnetically stirred for 2 hours, its temperature rose from 0 °C to room temperature. Then the solid residue was decanted twice and filtered off. The residue was dried on Petri dish and the samples were labelled accordingly as TSM-5ml-NaOH (corresponding to 0.07 mol), TSM-7.5ml-NaOH (corresponding to 0.14 mol), and TSM-10ml-NaOH (corresponding to 0.21 mol).

Total content of alkali metals in prepared materials was determined by dissolving of 0.1 g of sample in 50 ml of concentrated HNO_3_. Then, the solution was analysed *via* atomic absorption spectroscopy (AAS, Varian AA240FS) and the total contents of the alkali metal cations are shown in Table 1.

**Table 1:** Sample preparation details

Sample name	Amount of alkali (g)	Amount of alkali (mol)	pH of resulting suspensions	Content of Na (mg l^−1^)	Content of Na (mmol l^−1^)
TSM-5ml-NaOH	2.87	0.07	9	2.2	0.096
TSM-7.5ml-NaOH	4.31	0.14	10	133.8	5.817
TSM-10ml-NaOH	5.75	0.21	11	134.5	5.840

### 2.2. Characterization of the product

The following methods were used for morphological, structural, and chemical characterization of the product: scanning electron microscopy (SEM), transmission electron microscopy (TEM), surface area measurements (BET), thermal analysis (TG/DTA) and X-ray photoelectron spectroscopy (XPS). Details including experimental conditions are described in ESI – Characterization methods.†

### 2.3. Sorption experiments

Sorption of Cs(i), Sr(ii), Co(ii) and Eu(iii) on prepared titanates were investigated by batch sorption technique. The suspensions of 0.01 g of the materials and 1.5 ml of solutions of corresponding pH and ionic strength were first pre-equilibrated in test tubes for 24 h. The pH of solutions was adjusted with NaOH or HClO_4_ and the ionic strength was set to 0.01 or 0.1 mol l^−1^ by addition of NaClO_4_.

Kinetics of the uptake of Cs(i), Sr(ii),Co(ii) and Eu(iii) on the prepared samples were performed under following conditions: 1 × 10^−3^ mol l^−1^ CsCl at pH = 7.5, 1 × 10^−3^ mol l^−1^ SrCl_2_ at pH = 7.1, 1 × 10^−3^ mol l^−1^ CoCl_2_ at pH = 8.3 and 1 × 10^−3^ mol l^−1^ Eu(NO_3_)_3_ at pH = 8.4; *I* = 0.01 mol l^−1^ NaClO_4_, *V*/*m* = 150 ml g^−1^, *T* = 25 °C.

For sorption experiments, the radionuclide solutions labelled with ^134^Cs, ^85^Sr, ^60^Co and ^152–154^Eu radiotracer (^134^Cs, ^60^Co, ^152–154^Eu isotopes (POLATOM, Poland)), ^85^Sr isotope (PerkinElmer, USA) were added into the pre-equilibrated suspensions of materials and set for a specified time on a horizontal shaker (250 rpm, 25 ± 0.1 °C). A liquid-to-solid ratio was 150 ml g^−1^ for all experiments. The suspensions were filtered using mixed cellulose filters (pore size 0.2 μm) after shaking and aliquots of the filtrate (0.5 ml) were measured on the automatic gamma counter Wallac 1480 WIZARD 3 (PerkinElmer Life Sciences, Wallac Oy) with NaI(Tl) well-type detector. Each experiment was performed twice and the mean values are presented.

In order to investigate the influence of the pH on the sorption of selected radionuclides, the experiments were carried out in the pH range from ∼2 to ∼10 at ionic strength of 0.01 mol l^−1^. The pH of the solutions was measured using pH meter (Boeco BT-675) after equilibrium time of 24 h.

The sorption of radionuclides between the aqueous phase and the material was expressed as the uptake (%) and calculated by eqn (1):1
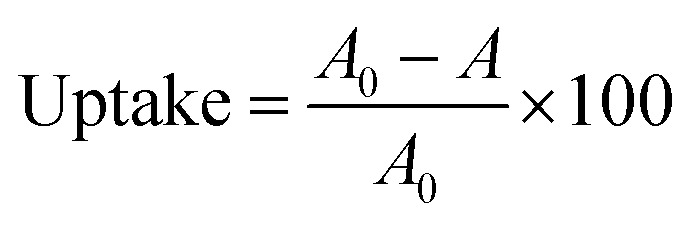
where *A*_0_ and *A* are values of relative activities of the solution of isotopes before and after their contact with the material.

For the determination of the above mentioned sorption isotherms for radionuclides on prepared samples, solutions of various concentration of CsCl, SrCl_2_, CoCl_2_ and Eu (NO_3_)_3_ (2.5 × 10^−5^ to 4 × 10^−2^ mol l^−1^) labelled by radiotracers were prepared. Contact time was 24 h.

The sorption isotherms were plotted as dependencies of equilibrated concentrations of radionuclides on materials on their equilibrated concentrations in liquid phase. The equilibrium concentrations of radionuclides in liquid and solid phases, *c*_eq_ (mol l^−1^) and *q*_eq_ (mmol g^−1^), respectively, were calculated as follows:2
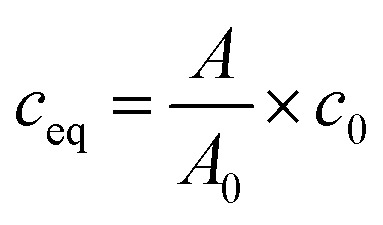
3
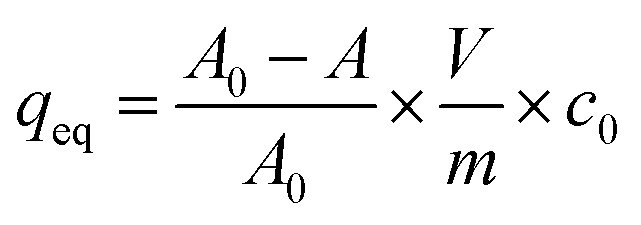
where *m* is mass of material (g), *V* is volume of liquid phase (mL), *A*_0_ and *A* are values of relative activities of the solution before and after equilibrating, *c*_0_ is the initial concentration of radionuclides in the solution (mol l^−1^).

The adsorption equilibrium data were fitted by Freundlich and Langmuir adsorption isotherms. The Freundlich model can be expressed by eqn (4):

4 *q*_eq_ = *K*_F_ × *c*^1/*n*^_eq_

where *K*_F_ (mmol g^−1^) and *n* are characteristic constants related to the adsorption capacity and adsorption intensity.

The Langmuir model is a theoretical model use for monolayer adsorption and can be described by eqn (5):5
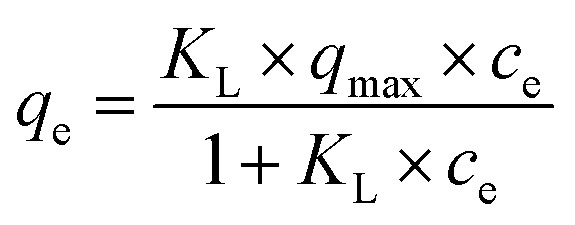
where *q*_max_ (mmol g^−1^) is the maximum adsorption capacity and *K*_L_ (l mmol^−1^) is a constant rated to the affinity of the binding sites.

The pseudo-first-order kinetic equation is (6):

6 *Q*_*t*_ = *Q*_e_(1 − e^−*k*_1_*t*^)

where *Q*_*t*_ is the amount absorbed in mg g^−1^ at time *t* in min, *Q*_e_ is the equilibrium adsorption capacity in mg g^−1^, and *k*_1_ is the rate constant in min^−1^.

## 3. Results and discussion

### 3.1. Prepared sorbents characterization

#### 3.1.1. Morphology

**Fig. 1:**
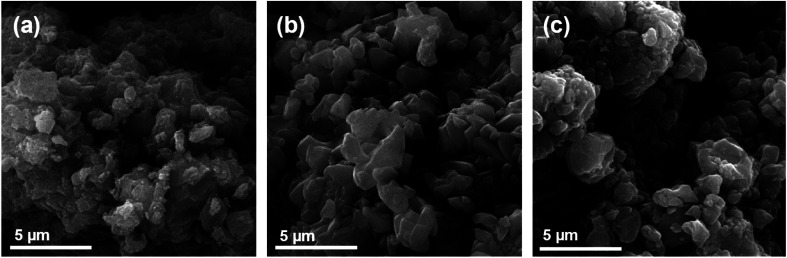
SEM of prepared samples TIG-5ml-NaOH (a), TIG-7.5ml-NaOH (b) and TIG-10ml-NaOH (c).

As already described, the starting titanyl sulphate monohydrate is formed by aggregates of isometric crystals with a broad size distribution. The EDS analysis shows the presence of S, O and Ti and their ratio is in line with the expected values based on the chemical formula.^19^ The controlled hydrolysis did not change the crystals of titanyl sulphate and these provided, after immersion into various concentrations of aqueous solution of sodium hydroxide, materials composed of aggregates of irregular planar crystals of size 1–2 μm (Fig. 1). The increasing amount of sodium hydroxide used during the preparation did not have any observable effect on the shape and size of the particles. Sufficient washing procedure of the prepared sorbents was confirmed by the EDS analysis with no traces of sulphur detected. The analysis also confirms the hypothesis that the higher the concentration of NaOH solution, the higher the amount of Na^+^ ions in the structure of the material with maximum concentration at 14%. Samples were clear of any detectable impurities (as analysed by the EDS method) in concentrations (approximately ≥0.01 wt%).

**Fig. 2:**
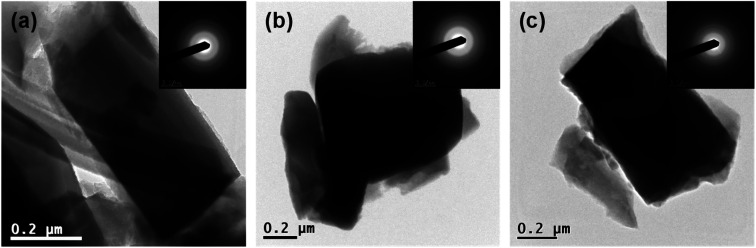
TEM/ED observations of prepared samples TIG-5ml-NaOH (a), TIG-7.5ml-NaOH (b) and TIG-10ml-NaOH (c).

Detailed investigation of prepared materials was conducted *via* transmission electron microscopy where individual crystals were observed (Fig. 2). Only wide indefinite rings were discovered in the SAED patterns (inset Fig. 2) implying amorphous state of all the prepared samples.

#### 3.1.2. Sorbents behaviour under heating

**Fig. 3:**
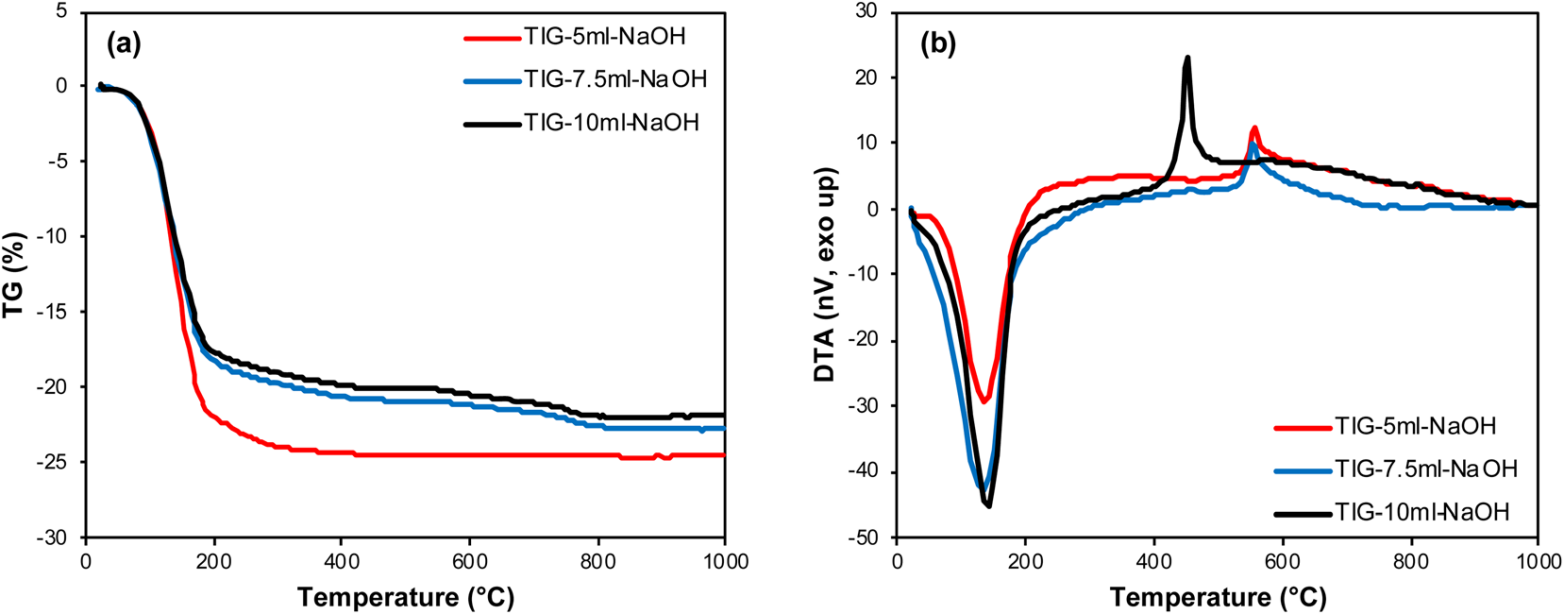
Comparison thermal behaviour of prepared samples – TG (a) and DTA (b).

The process of decomposition of samples TIG-5ml-NaOH and TIG-10ml-NaOH was previously investigated in ref. 23. The decomposition process of the sample TIG-7.5ml-NaOH leads to a total mass loss of 20–25% (Fig. 3a). During the first decomposition step, which is endothermic, water and carbon dioxide evolve. The second decomposition step is characterized by an exothermic peak with a maximum at 450–580 °C related to the observable crystallization of amorphous phase (Fig. 3b). The data then confirms the similarity between the samples precipitated by 7.5 ml and 10 ml which is described in further detail below.

#### 3.1.3. XPS analysis

The prepared samples were analysed by X-ray photoelectron spectroscopy (XPS) and the survey scan revealed the presence of oxygen, titanium, sodium and carbon. Adventitious carbon centred at 284.8 eV was used for calibration of the spectra. Superficial stoichiometry showed no amount of sodium present in the analysed surface of the sample TIG-5ml-NaOH (Table 2).

**Table 2:** XP superficial stoichiometry of prepared samples

Element	Elemental composition (at%)		
	TIG-5ml-NaOH	TIG-7.5ml-NaOH	TIG-10ml-NaOH
Ti 2p	0.107	0.138	0.145
O 1s	0.457	0.510	0.457
Na 1s	0	0.056	0.062
C 1s	0.436	0.297	0.337

**Fig. 4:**
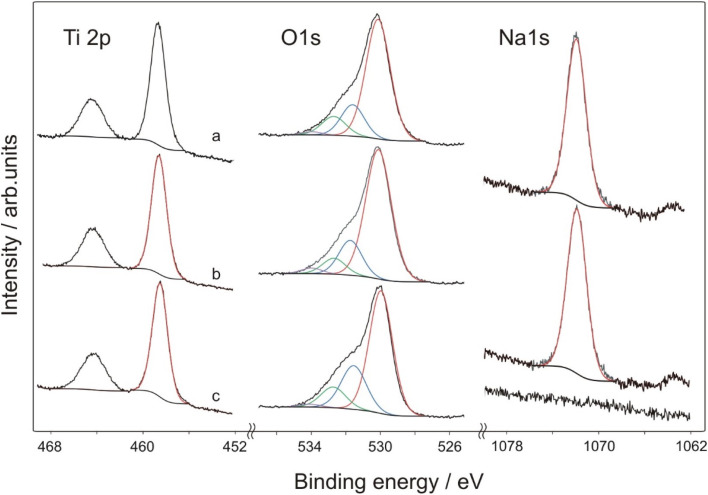
X-ray photoelectron spectroscopy (XPS) spectra of the prepared samples, (a) TIG-7.5ml-NaOH, (b) TIG-10ml-NaOH, (c) TIG-5ml-NaOH.

Titanium is present in the form of TiO_2_. Ti 2p_3/2_ peak is centred at 458.5 eV for the sample TIG-5ml-NaOH with no sodium observed in the superficial layer (Fig. 4). Separation of 2p_3/2_–2p_1/2_ is 5.75 eV, which is a typical value for titanium dioxide. In the samples TIG-10ml-NaOH and TIG-7.5ml-NaOH, the Ti 2p_3/2_ bands are centred at 458.4 and 458.3 eV respectively. Oxygen is present mainly in the metal-oxide lattice which is found at binding energies 530.0 eV in all samples. Contributions of C=O and M–OH (531.5 eV), C–O (532.7 eV) and adsorbed water (533.9 eV) were also revealed in all samples. Sodium is found in the superficial layer in both samples TIG-10ml-NaOH and TIG-7.5ml-NaOH, the band is relatively broad (FWHM = 1.9 eV) and positioned to 1071.9 eV.

After moderate sputtering, lower oxidation states Ti^3+^ oxide (2p_3/2_ = 457.1 eV) and Ti^2+^ oxide (2p_3/2_ = 455.6 eV) appeared. It is known, that argon ions reduce Ti^4+^ and lower oxidation states are observed as a result. Oxygen bands did not change by sputtering, while sodium bands were narrower by 0.2 eV. It is an evidence that sodium is present at least in two forms, with the most superficial one removed by sputtering.

Atomic ratios Ti/Na in the prepared samples were calculated to be 1.82 in the TIG-10ml-NaOH and 1.77 in the TIG-7.5ml-NaOH, while sputtered samples were sodium depleted (Ti/Na = 2.48 in the TIG-10ml-NaOH and 2.32 in the TIG-7.5ml-NaOH). It showed that sodium is present predominantly at the surface.

In the literature, the role of sodium doping is discussed. Some authors highlight the bigger atomic radius of Na^+^ (1.02 Å) in comparison with Ti^4+^ (0.68 Å) and demonstrate difficult substitutional doping and expect migration of Na^+^ to TiO_2_ surface.^24,25^ In our samples, sodium was found partially depleted after moderate argon ion sputtering, which supports expected separation of Na^+^ on TiO_2_ nanoparticles. In the recent paper,^26^ authors observed shift in the position of Ti^4+^ band to lower binding energies and explained it by substitutional doping of Na^+^ at Ti^4+^ site. In our samples we also observed shifted position of Ti 2p_3/2_ from 458.5 eV (no sodium present) to 458.4 (lower Na content) and 458.3 eV (higher Na content), which can be an evidence of partial sodium involvement in Ti–O network.

#### 3.1.4. Surface area determination

**Fig. 5:**
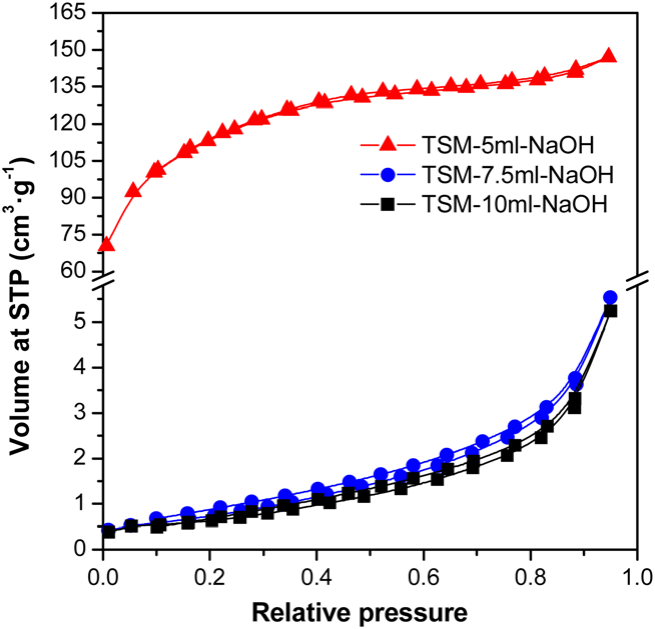
Adsorption/desorption isotherms of all prepared samples.

The results of the surface area and porosity measurements (Fig. 5) demonstrate mostly microporous character of the sample TSM-5ml-NaOH with external surface area 82 m^2^ g^−1^and micropore surface area 329 m^2^ g^−1^. In contrast, the purely mesoporous character of samples can be observed for TSM-7.5ml-NaOH with BET surface area 2.8 m^2^ g^−1^ and TSM-10ml-NaOH with BET surface area 2.3 m^2^ g^−1^. The pore volume in the mesoporous parts was 0.040 cm^3^ g^−1^ for the sample TSM-5ml-NaOH, in the higher base addition samples, the pore volume was 0.008 cm^3^ g^−1^. The pore volume in microporous parts was 0.177 cm^3^ g^−1^. Average pore diameter in mesoporous parts was 3.5 nm and in microporous 0.45 nm.

**Fig. 6:**
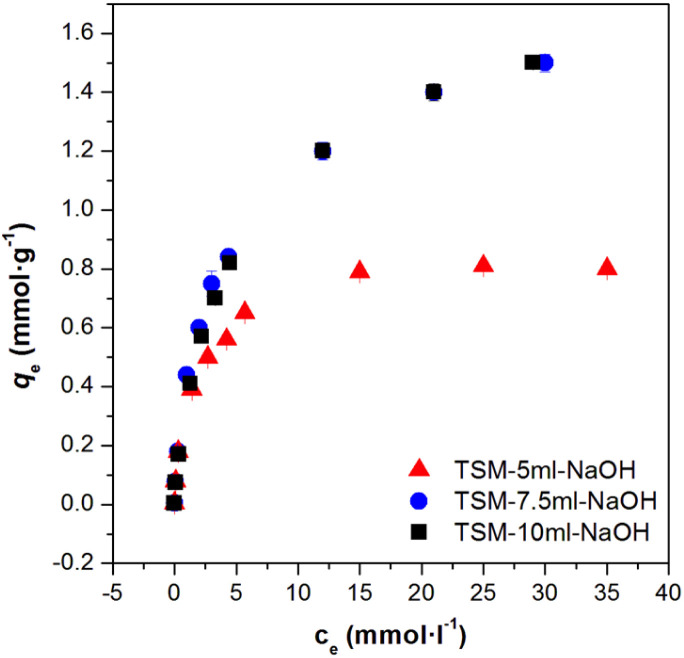
Comparison of the maximum sorption capacities for Cs(i) on selected samples.

Samples with the addition of 7.5 ml, or 10 ml of NaOH had a significantly lower surface area than samples with the addition of 5 ml of NaOH, and according to the shape of the isotherm, the samples were completely mesoporous. A likely explanation for such a large reduction in the value of surface area could be the blockage of the micropores of the material by Na^+^ ions. This statement is also supported by the fact that for samples that were precipitated with another base (aqueous ammonia), such a rapid decrease in surface area was not observed (Table 3). Moreover, it is also evident from Fig. 6 that the samples with a low value of the measured surface area did not show lower sorption capacities and this is because the surface area is probably not that low, it is just effectively blocked by Na^+^ ions, which make correct surface area measurement impossible.

**Table 3:** Values of the surface area measurements for different alkalis used

Sample name	Surface area (m^2^ g^−1^)
TSM-5ml-NaOH	411
TSM-7.5ml-NaOH	3
TSM-10ml-NaOH	2
TSM-5ml-NH_3_	299
TSM-7.5ml-NH_3_	189

The specific surface area and pore volume parameters characterizing various titanate structures have been collected in ref. 1. It was described that the layered compounds, and titanate nanotubes in particular, possess specific surface areas higher than those of silicotitanates. The specific surface areas of nanofibers, nanoribbons and nanowires are of a comparable magnitude but they are smaller than those of nanotubes.

The total content of alkali metal in the prepared materials was obtained as follows: 0.1 g of the sample was dissolved in the concentrated HNO_3_ under heating with the final solution volume at 50 ml. Solution analysis was then performed *via* atomic absorption spectroscopy with the detected total content of sodium in prepared samples being 0.05 mmol g^−1^ for TSM-5ml-NaOH, 2.90 mmol g^−1^ for TSM-7.5ml-NaOH and 2.92 mmol g^−1^ for TSM-5ml-NaOH.^23^ The significant differences in Na content could also justify the differences in surface area (of prepared samples) as the pores available for nitrogen adsorption could be blocked by the sodium ions.^23^

### 3.2. Application of prepared samples as sorbents of radionuclides

#### 3.2.1. Samples' pre-testing

From a comparison of the maximum sorption capacities for Cs(i) on TSM 5ml-NaOH (113.9 mg g^−1^) and on TSM-7.5ml-NaOH (206.3 mg g^−1^) sorbents, it is evident that the assumption of a higher specific surface area providing a higher sorption capacity has not been confirmed (Fig. 6), the prepared sample TSM-7.5ml-NaOH is an excellent sorbent of caesium ions and the adsorbed amount is not dependent on the surface area of the samples but on the amount of sodium ions.

Based on these results, the adjusted preparation method of TSM-7.5ml-NaOH has been chosen as the most suitable and is used in the following experiments for water waste treatment.

#### 3.2.2. Kinetics studies

**Fig. 7:**
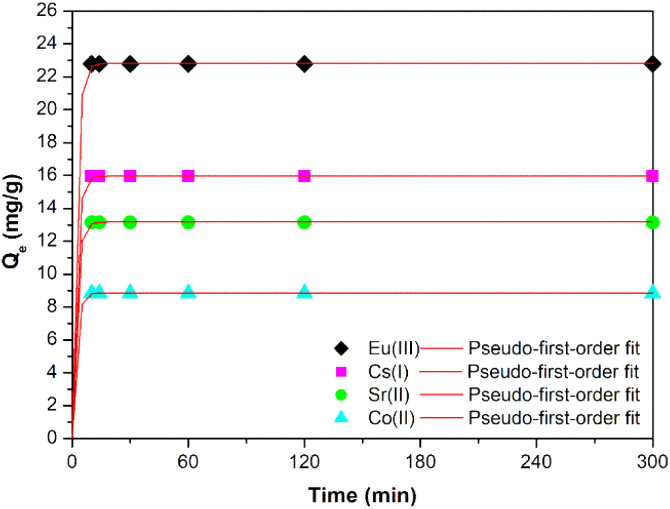
Kinetics of the uptake of Cs(i) and Sr(ii), Co(ii) and Eu(iii) on the prepared sample; with pseudo-first-order model.

Fast removal is particularly desirable in emergency situations, therefore, first experiments studied on selected sample TIG-7.5ml-NaOH were the uptake kinetics of selected radioactive ions. The influence of contact time on Cs(i), Sr(ii), Co(ii) and Eu(iii) uptake on prepared sample (Fig. 7) shows that the equilibrium is reached in less than 10 minutes after the initial contact between sorbent and Sr(ii), Co(ii) and Eu(iii) confirming its quick uptake capabilities compared to^5,27^. The slowest kinetics of uptake were observed with Cs(i) ions with equilibrium at 30 minutes and probably it was slowed by the competing Na(i) ions present in the sample. At equilibrium, the Cs(i) removal reached 83%, the Sr(ii), Co(ii) and Eu(iii) reached 100% at the conditions described in Table 4. Fig. 7 shows the fitting of the adsorption kinetics of the prepared sample with the pseudo-first-order equation. The corresponding fitting parameters are presented in Table 5. The adsorption kinetics were also fitted with a pseudo-second order equation, but the coefficients of determination (*R*^2^) were lower in all cases.

**Table 4:** Conditions of measurements used in kinetics experiments at *I* = 0.01 mol l^−1^, *T* = 25 °C, *V*/*m* = 150 ml g^−1^

Radionuclide	Used salt	concentration (mmol l^−1^)	pH
Cs(i)	CsCl	1 × 10^−3^	7.5
Sr(ii)	SrCl_2_	1 × 10^−3^	7.1
Co(ii)	CoCl_2_	1 × 10^−3^	8.3
Eu(iii)	Eu(NO_3_)_3_	1 × 10^−3^	8.4

**Table 5:** Kinetic parameters for metal sorption on the prepared sample

Radionuclide	*k*_1_ (min^−1^)	*Q*_e_ (mg g^−1^)	*R* ^2^
Cs(i)	0.486	15.967	0.984
Sr(ii)	0.499	13.137	0.986
Co(ii)	0.503	8.860	0.989
Eu(iii)	0.484	22.822	0.985

#### 3.2.3. Effect of pH and ionic strength

**Fig. 8:**
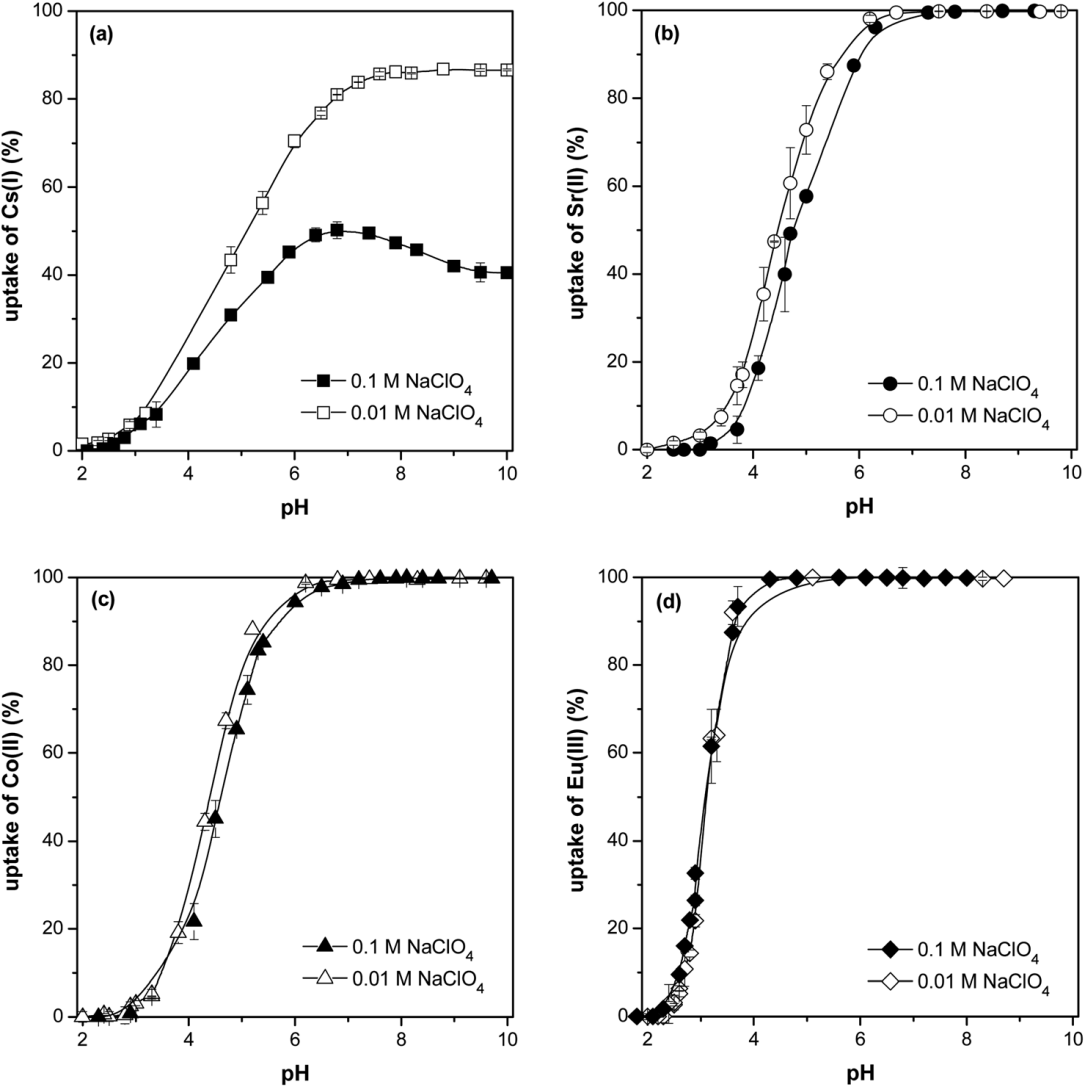
The effect of equilibrium pH on Cs(i) (a), Sr(ii) (b), Co(ii) (c) and Eu(iii) (d) uptake on prepared material (various ionic strength, *V*/*m* = 150 ml g^−1^, contact time 24 h, *T* = 25 °C); the error bars represent deviations.

The influence of pH equilibrium and ionic strength (*I*) on sorption of Cs(i), Sr(ii), Co(ii) and Eu(iii) by the sorbent is demonstrated in Fig. 8. The pH of the solutions is a very important parameter of sorption process as it has a great influence on the speciation of metals, protonation degree of oxygen-containing functional groups on material's surfaces (hydroxyl, carboxyl groups) and competition of metal ions for the binding site. Ionic strength is the total ion concentration in solution. It is a property governing the shielding of charges in solution. Natural water bodies generally contain some level of dissolved salts which in turn translate to their ionic strength.

It is clear, based on the obtained data with *I* = 0.01 mol l^−1^ NaClO_4_, that the higher the pH, the higher the sorption of Cs(i), Sr(ii), Co(ii) a Eu(iii) until a maximum is reached when the sorption stabilizes. Maximum sorption (quantitative ≥ 99.9%) was reached at pH ≥ 7.5 for Cs(i), pH ≥ 6.5 for Sr(ii) and Co(ii) and pH ≥ 4.5 for Eu(iii). At pH = 6, the ion affinity towards the sorbent decreases in agreement with the decreasing ion potential in the following order: Eu(iii) > Sr(ii), Co(ii) > Cs(i).

**Fig. 9:**
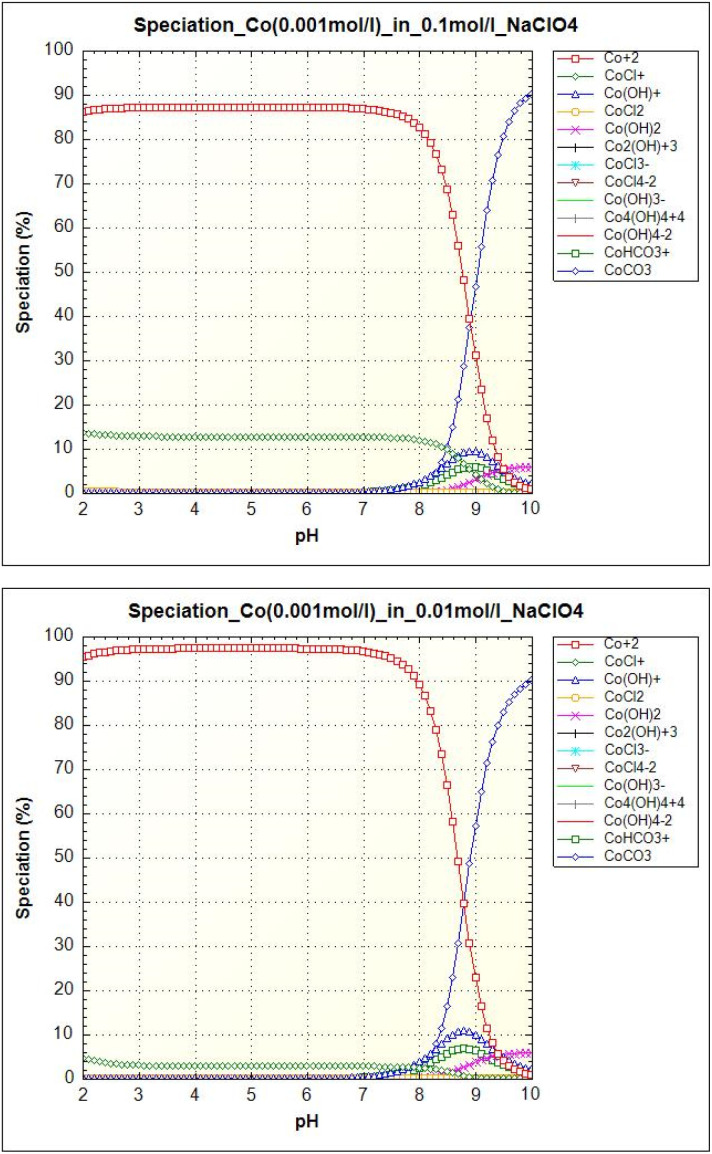
Cobalt (0.001 mol l^−1^) speciation as a function of pH in an aqueous solution with ionic strength 0.1 mol l^−1^ NaClO_4_ (left) and 0.01 mol l^−1^ NaClO_4_ (right).

The ionic species of Co(ii) and Eu(iii) in solution with different pH values and ionic strengths are different. Co(ii) and Eu(iii) speciations were modelled by PHREEQC program. Speciation of cobalt is shown in Fig. 9. The majority species of free cobalt ions (Co^2+^) is present for pH < 8. Carbonate form CoCO_3_ appears at pH values above 8. At higher ionic strength (0.1 mol l^−1^), chloride phase CoCl^+^ occurs up to pH 9.5. The minor speciations (Co(OH)^+^, Co(HCO_3_)) are observed for pH values between 7.5 and 10.0 and hydroxide form Co(OH)_2_ is visible above pH 9.0. It is clear from the equilibrium pH on Co(ii) uptake dependencies that the sorbent adsorbed all of the above cobalt speciations.

**Fig. 10:**
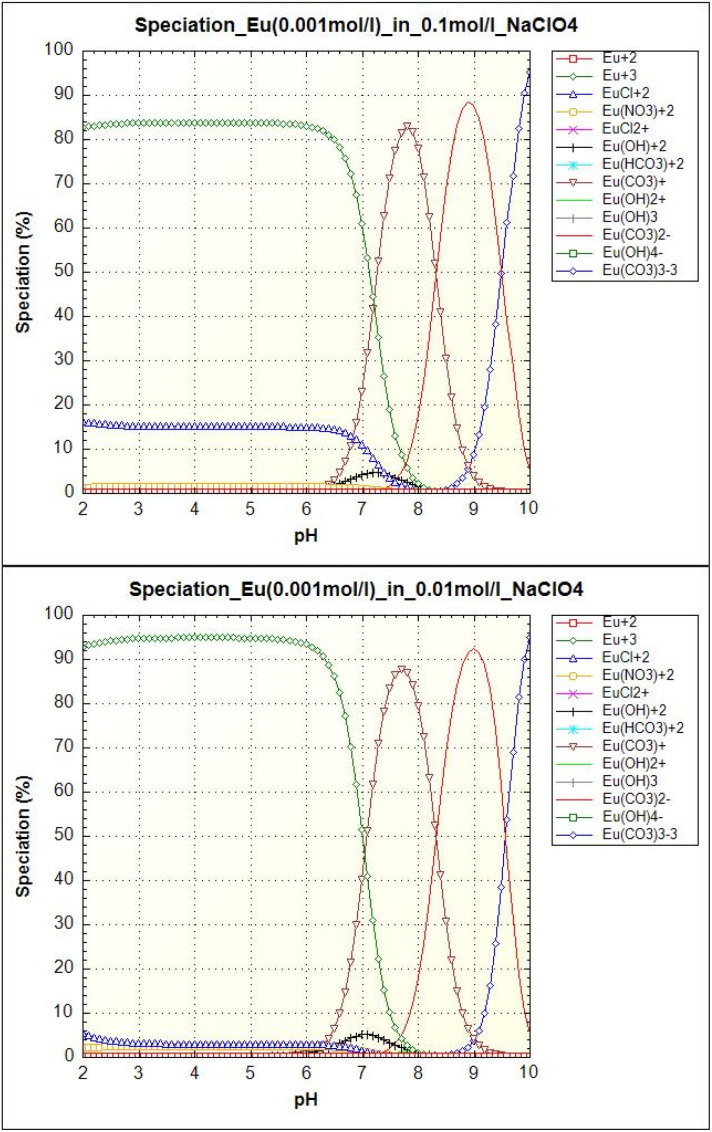
Europium (0.001 mol l^−1^) speciation as a function of pH in an aqueous solution with ionic strength 0.1 mol l^−1^ NaClO_4_ (left) and 0.01 mol l^−1^ NaClO_4_ (right).

As we can see in Fig. 10, europium can be present in free form (Eu^3+^), complexed form with dissolved carbonates (Eu(CO_3_)^+^, Eu(CO_3_)_2_^−^, Eu(CO_3_)_3_^3−^), chloride ions (EuCl_2_^+^), or in hydrolyzed form (Eu(OH)_2_^+^). Under our conditions, the majority species is the Eu^3+^ ion for pH < 6.5. For pH values between 6.5 and 8.5, Eu(CO_3_)^+^ species predominates. Other carbonate species Eu(CO_3_)_2_^−^ and Eu(CO_3_)_3_^3−^ appear at pH values above 8.5. The smaller hydrolyzed form Eu(OH)_2_^+^ occurs at pH 6.5 to 8.0. In the case of higher ionic strength (0.1 mol l^−1^), chloride phase EuCl_2_^+^ occurs up to pH 7.5. On the basis of the equilibrium pH data on Eu(iii) uptake, it can be concluded that the sorbent adsorbed all europium forms.

Fig. 8 also shows, that the effect of ion strength on sorption is most noticeable with Cs(i) ions, with approx. twofold sorption under low *I* (0.01 mol l^−1^ NaClO_4_) when compared to sorption under higher *I* (0.1 mol l^−1^ NaClO_4_) in range of pH 7–10 which is in good agreement with data published in ref. 28. The Sr(ii) and Co(ii) uptakes are lightly affected by the Na(i) ions in pH range 2–6.5, there is no *I* effect on sorption with higher pH. No effect of *I* on Eu(iii) was observed at sorption throughout the pH range. Generally, the surface complexation and/or ion exchange is susceptible to exposure to ion forces, while the inner surface complexation is not influenced by the ion forces.

#### 3.2.4. Adsorption isotherms

**Fig. 11:**
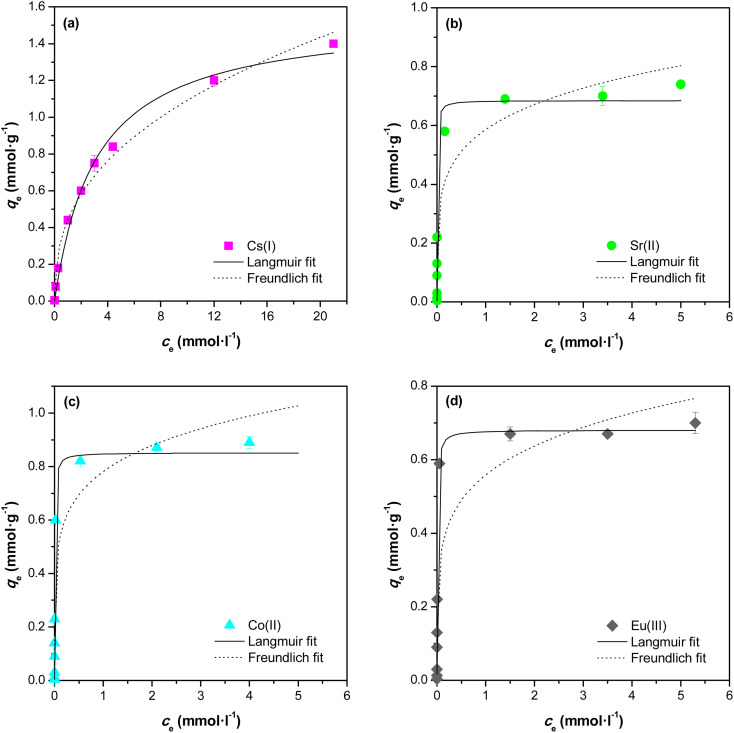
Experimental data fitted to Langmuir and Freundlich isotherms for sorption of Cs(i), Sr(ii), Co(ii) and Eu(iii) (various concentrations of CsCl at pH = 9.2 or SrCl_2_ at pH = 7.2 or CoCl_2_ at pH = 6.9 or Eu(NO_3_)_3_ at pH = 6.2; I = 0.01 mol l^−1^ NaClO_4_, *V*/*m* = 150 ml g^−1^, contact time 24 hours, *T* = 25 °C; deviations represent confidence interval (*α* = 0,05)).

The sorption isotherms of Cs(i), Sr(ii), Co(ii) a Eu(iii) on the prepared material are shown in Fig. 11. Sorption isotherms were detected in pH 6–9, *i.e.* in high sorption range of studied ions. For modelling experimental equilibrium data, both Freundlich and Langmuir isotherm models were used. The reliability comparison clearly shows that the Langmuir isotherm is a more suitable fit than the Freundlich isotherm for ion sorption in samples, suggesting mononuclear ion sorption on the material. As calculated from the Langmuir model, the maximum amount of adsorbed ions equalled to 206.3 mg g^−1^ for Cs(i), 60.0 mg g^−1^ for Sr(ii), 50.2 mg g^−1^ for Co(ii) and 103.4 mg g^−1^ for Eu(iii) (Table 6). The calculated capacities in mg of adsorbed amount of radionuclide per gram of sorbents were compared to nanotube titanates (72 mg Sr(ii) g^−1^, resp. 199 mg Cs(i) g^−1^)^1,3,6,29^ or nanofiber titanates (55 mg Sr(ii) g^−1^, resp. 66 mg Cs(i) g^−1^),^1,4,30,31^ manganese oxide (101.1 mg Sr(ii) g^−1^, resp. 68.2 mg Cs(i) g^−1^),^1,4,30,31^ K_2_[CoFe(CN)_6_] (39.6 mg Cs(i) g^−1^)^32,33^ or biogenic hydroxyapatite (13.2 mg Sr(ii) g^−1^).^34^ The high capacity of prepared samples significantly exceeds published data obtained with related materials and are, therefore, considered as very promising for use as sufficient sorbent of radionuclides.

**Table 6:** Freundlich and Langmuir parameters data for the sorption of radionuclides from aqueous solutions by prepared sorbent

radio-nuclide	Freundlich model			Langmuir model		
	*K*_F_ [mmol g^−1^]	*n*	*R* ^2^	*q*_max_ [mg g^−1^]	*K*_L_ [l mmol^−1^]	*R* ^2^
Cs(i)	0.442	2.54	0.985	206.3	0.318	0.991
Sr(ii)	0.586	5.10	0.930	60.0	203.0	0.988
Co(ii)	0.781	5.87	0.922	50.2	164.3	0.970
Eu(iii)	0.559	5.26	0.841	103.4	140.1	0.973

#### 3.2.5. Mapping of samples after sorption

A series of experiments with non-active radionuclides (the samples were not labeled with radiotracer) was performed simultaneously with active radionuclides (the samples were labeled with radiotracer) for purposes of mapping of samples by TEM/EDS analysis after adsorption tests and SEM/EDS analysis.

**Fig. 12:**
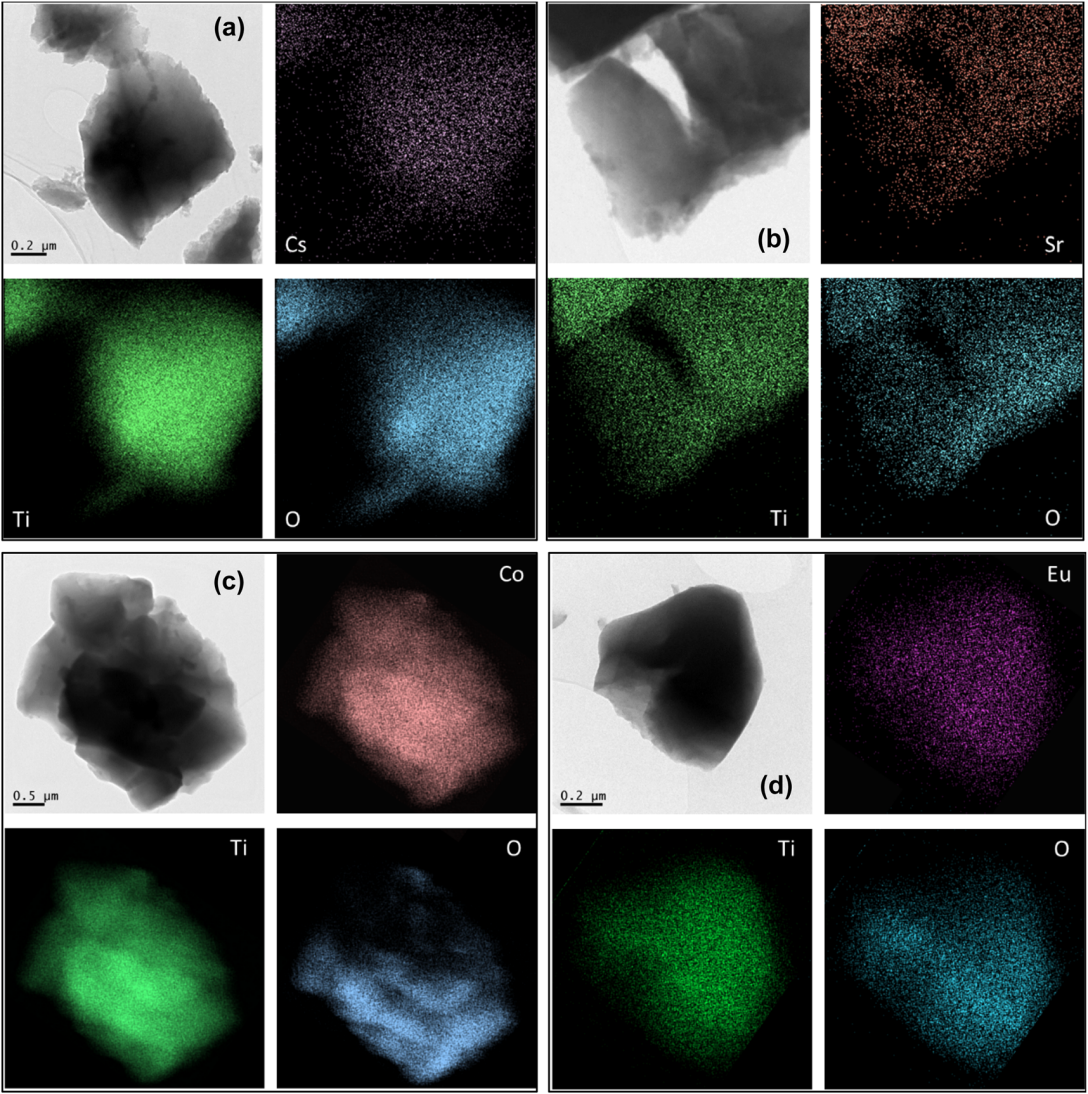
TEM/EDS mapping of O, Ti and radionuclide in prepared sample after adsorption of Cs(i) (a), Sr(ii) (b), Co(ii) (c) and Eu(iii) (d).

It can be seen in Fig. 12 that the radionuclides are homogenously distributed in the sample with no obvious location preference.

With the goal of proving an ion exchange theory, the samples after sorption were analyzed by SEM/EDS analysis and the results are summarized in Table 7. The variance in the adsorbed amount of Na ions would indicate that the mechanism of sorption is ion exchange as is in good agreement with data published earlier.^8,11^

**Table 7:** EDS analysis of samples after adsorption experiments

Sorption of radionuclide	Exchanged amount of radionuclide (wt%)	Remained amount of sodium ions (wt%)
Cs(i)	6.4	<0.01
Sr(ii)	3.1	<0.01
Co(ii)	3.0	<0.01
Eu(iii)	2.2	<0.01

We can summarize that the sorbent prepared according to the described procedure is highly effective and has all the prerequisites for technological application. The synthetic process itself does not use environmentally harmful raw materials and can be easily connected to the sulphate technology of titanium white production. Its price in commercial preparation should therefore not exceed the price of common types of white titanium pigments and should be significantly cheaper than zeolite based materials currently considered as very promising.

## 4. Conclusions

Three novel titanates were synthesized, characterized and studied for the removal of Cs(i), Sr(ii), Co(ii) and Eu(iii) from aqueous environment. The comparison of sorption capacities of TIG-5ml-NaOH, TIG-7.5ml-NaOH and TIG-10ml-NaOH disproved the assumption that the higher the surface area the higher sorption capacity. The results showed a rapid removal kinetics (up to 10 minutes) and a high equilibrium capacity for selected sorbent, the maximum of adsorbed amounts were calculated from the Langmuir model, the maximum amount of adsorbed ions equalled to 206.3 mg g^−1^ for Cs(i), 60.0 mg g^−1^ for Sr(ii), 50.2 mg g^−1^ for Co(ii) and 103.4 mg g^−1^ for Eu(iii). The removal mechanism is most likely ion exchange followed by complexation reactions. The results showed that the prepared materials exhibit much faster kinetics than commercial adsorbents.

Combining the results of adsorption experiments with material characteristics leads to a conclusion that (for studied titania based adsorbents) the radionuclides adsorption efficiency increases with increasing amount of present exchangeable cations.

## Conflicts of interest

There are no conflicts to declare.

## Supplementary Material

RA-014-D3RA05663E-s001
