# Supplementary material for: Highly efficient eco-friendly sodium titanate sorbents of Cs(i), Sr(ii), Co(ii) and Eu(iii): synthesis, characterization and detailed adsorption study
Source: RSC Adv. 2024 Jan 5;14(1):743–54. doi: 10.1039/d3ra05663e (PMC10768530; doi:10.1039/d3ra05663e)
Supplement: RA-014-D3RA05663E-s001 [file RA-014-D3RA05663E-s001.pdf]

Electronic Supplementary Material (ESI) for RSC Advances.
This journal is © The Royal Society of Chemistry 2024

Highly efficient eco-friendly sodium titanate sorbents of Cs(I), Sr(II), Co(II) and Eu(III): Synthesis, characterization and detailed adsorption study.

Monika Motlochová[1,2], Lórant Szatmáry*[3], Eva Pližingrová[1], Petra Salačová[3], Radek Fajgar[4], Sven Lidin[2], Jan Šubrt[1]*

[1]Institute of Inorganic Chemistry of the Czech Academy of Sciences, 250 68 Řež, Czech Republic

[2]Centre for Analysis and Synthesis, Lunds Universitet, Naturvetarvägen 14, Lund 222-61, Sweden

[3]Fuel Cycle Chemistry Department, UJV Rez a.s., 250 68 Řež, Czech Republic

[4]Institute of Chemical Process Fundamentals of the Czech Academy of Sciences, Rozvojova 135, Prague 160 00, Czech Republic

*motlochova@iic.cas.cz, lorant.szatmary@ujv.cz

Characterization methods

The following methods were used for morphological, structural, and chemical characterization of the product: scanning electron microscopy (SEM), transmission electron microscopy (TEM), surface area measurements (BET), thermal analysis (TG/DTA) and X-ray photoelectron spectroscopy (XPS).

Structural and morphology characterization of the samples were observed by scanning electron microscopy (SEM). A Philips XL 30 CP microscope (W-cathode, 20 nm resolution at 1 kV) was used for initial observation.

Detailed phase analysis including imaging and electron diffraction were carried out on a transmission electron microscope (TEM) JEOL JEM 3010 operated at 300 kV (LaB6, cathode, point resolution 1.7 Å). Images were recorded on a Gatan CCD camera with resolution of 1024 × 1024 pixels using the Digital Micrograph software package. The powder samples were dispersed in ethanol and the suspension was treated in ultrasound for 2 min. A drop of very

dilute suspension was placed on a holey-carbon coated Cu-grid and allowed to dry by evaporation at ambient temperature.

Thermogravimetry (TG), differential thermal analysis (DTA) and evolved gas analysis (MS) were carried out in apparatus SetSys Evolution (SETARAM). Thermoanalytical measurements were carried out in an inert atmosphere (argon, 60 cm3 min-1), in an open crucible made of a-Al2O3, and initial sample mass was approximately 20 mg (crushed to fine powders). Samples were heated from the room temperature to 1000 °C with heating rate of 10 °C min-1 which enabled a detailed study of the whole decomposition process.

Surface area was determined by the BET method using a Quantachrome Nova 4200e instrument. Nitrogen adsorption was car- ried out at −196 °C. Before analysis, the non-annealed samples were pretreated at RT under vacuum for 35 h.

The XPS spectra were obtained by Kratos ESCA 3400 with base pressure lower than 5.0 10-7 Pa, using polychromatic Mg X-ray source (Mg Ka, 1253.4 eV). Spectra were taken over Ti 2p, O 1 s, C 1 s and N 1 s region. Every sample was measured twice, i.e., samples with no special treatment and samples after Ar? ions sputtering (1.5 kV, 90 s) in order to remove surface contamination. Powders were fixed on pins using a carbon tape. The overlapping spectral features were resolved into individual components using damped nonlinear least squares method and the lines of Gaussian–Lorentzian shape. Prior to fitting, the Shirley background had been subtracted.
